# Zoonotic potential of ESBL-producing coliforms in pastorally managed ruminants with subclinical mastitis in Plateau State, Nigeria

**DOI:** 10.3389/frabi.2025.1632264

**Published:** 2025-09-15

**Authors:** Kenneth Nnamdi Anueyiagu, Ebere Roseann Agusi, Dennis Kabantiyok, Grace Mebi Ayanbimpe, Eugene Ifeanyichukwu Ikeh

**Affiliations:** ^1^ Federal College of Animal Health and Production Technology, National Veterinary Research Institute (NVRI) Vom, Vom, Plateau State, Nigeria; ^2^ Biotechnology Division, National Veterinary Research Institute (NVRI), PMB 01 Vom, Vom, Plateau State, Nigeria; ^3^ Fleming Laboratory, Diagnostic Services Department, National Veterinary Research Institute (NVRI), PMB 01 Vom, Vom, Plateau State, Nigeria; ^4^ Department of Medical Microbiology, University of Jos, Jos, Nigeria

**Keywords:** subclinical mastitis, ESBL-producing coliforms, antimicrobial resistance, one health, zoonosis, ruminants, Nigeria

## Abstract

**Background:**

Environmental coliform bacteria are frequently the cause of subclinical mastitis (SCM), a serious health issue in the dairy industry. Extended-spectrum β-lactamase (ESBL)-producing coliforms in livestock are a serious public health concern, particularly in environments where people and animals coexist. With an emphasis on their zoonotic and One Health implications, this study sought to evaluate the incidence of SCM and the occurrence of ESBL-producing coliforms in ruminants in Plateau State, Nigeria.

**Methods:**

The California Mastitis Test (CMT) was used to screen 287 milk samples that were taken from cows, ewes, and does. Standard microbiological methods were used to identify the bacterial isolates from CMT-positive samples. The presence of resistance genes (*bla*_TEM_ and *bla*_CTX-M_) was ascertained by PCR, and ESBL production was confirmed phenotypically. Phylogenetic analysis showed genetic diversity and possible horizontal gene transfer among isolates.

**Results:**

Out of 287 milk samples, 79 (27.5%) had subclinical mastitis through the CMT, with a higher prevalence recorded in does 18(22.8%) while ewes and cows recorded 23(29.1%), and 38(48.1%) respectively. Of the 79 CMT-positive samples, the following isolates were identified: *Citrobacter freundii* (6.3%), *Klebsiella pneumoniae* (21.6%), *K. oxytoca* (2.5%), *K. aerogenes* (6.3%), and *E. coli*, being the most prevalent in cows (71%). Through PCR, 46 isolates expressed two important ESBL genes, *bla*_TEM_ and *bla*_CTX-M._

**Conclusion:**

A possible zoonotic reservoir for antibiotic resistance in Nigeria is highlighted by the increased frequency of ESBL-producing coliforms in ruminants with SCM. These results highlight the necessity of implementing integrated One Health initiatives, such as public education, surveillance, and antimicrobial stewardship, in order to reduce the risk of resistant pathogen transmission from animals to people.

## Introduction

Mastitis, an inflammation of the mammary gland that is particularly common in sub-Saharan Africa, including Nigeria, is one of the most economically important conditions affecting the intrinsic quality of ruminant dairy products globally (Halasa et al., 2007; Abebe et al., 2022). Based on the clinical condition of the mammary gland, mastitis is classified into two groups: clinical and subclinical mastitis. Clinical mastitis presents with sudden redness and a painful udder that secretes low-quality milk with higher somatic cell counts (Cobirka et al., 2020). In contrast, subclinical mastitis is characterized by the absence of visible signs in the udder and does not visibly reveal the quality of poor milk (Ruegg, 2017).

Due to their opportunistic tendencies and ubiquitous nature in the environment, coliform bacteria, particularly *Escherichia coli* (*E.coli*), *Klebsiella* spp., and *Enterobacter* spp., are becoming increasingly implicated in both clinical and subclinical cases as causal agents (Bachaya et al., 2021). The rise of coliforms that produce extended-spectrum beta-lactamase (ESBL) in mastitic milk is a growing concern (Klibi et al., 2019). Particularly when this is connected with toxigenic pathogens such as *Staphylococcus aureus*, which produces enterotoxins in milk-based products, leading to toxic shock syndrome, food poisoning, and cramps (Galal et al., 2006). These coliforms are resistant to a variety of beta-lactam antibiotics, which limits the available treatment choices and poses serious health hazards to the general public (Iroha et al., 2023).

Because ESBL-producing coliforms can spread through direct contact with infected animals, ingestion of raw or inadequately pasteurized milk, and environmental pollution, their zoonotic potential is especially concerning (Tansawai et al., 2022). Antimicrobial-resistant bacteria can proliferate and spread throughout animal and human populations in Nigeria, mainly through the food chain and through direct contact with farmers in the smallholder and informal dairy systems. This is exacerbated due to inadequate veterinary supervision, poor biosecurity, excessive antibiotic usage, and unsanitary practices (Odetokun et al., 2020).

Mastitis in Nigerian livestock has been extensively studied in small ruminants, especially goat, with data on subclinical mastitis appearing in cattle in the late 2000s (Shittu et al. 2012). The disease has been described as common in Nigeria due to the farming system and poor hygiene around farms, which promote infection of the udder and subsequently low-quality milk and milk-based products. While several microbes have been implicated in the pathogenesis of mastitis, coliforms have been identified as the most notorious group of pathogens with a higher incidence of cow death and agalactia-related culling when compared to other pathogens (Mbuk et al., 2016). Food animals consume 69,455 tonnes of antimicrobials globally (Ardakani et al., 2024), and a significant portion of these antimicrobials goes into the treatment of mastitis in dairy animals (Theodoridou Oxinou et al., 2025). The excessive use of antibiotics in dairy animals is a major cause of antibiotic use in dairy animals. This is further compounded by the fact that up to 20% of subclinical mastitis (SCM) are unrelated to microbial infection (Mdegela et al., 2009), underscoring the need for caution in the administration of antibiotics.

In the context of ruminant mastitis, this study provides a critical framework for addressing the shared burden of antimicrobial resistance (AMR) caused by ESBL-producing pathogens (WHO, 2022). Understanding the prevalence, drivers, and health risks associated with these resistant coliforms in Nigerian livestock systems is essential for developing integrated strategies to safeguard both public health and animal productivity. This study aims to determine the zoonotic and one health implications of ESBL-Producing Coliforms in ruminants with Subclinical Mastitis in Plateau State, Nigeria.

## Materials and methods

### Data collection

This study was conducted in Jos North and Jos South Local Government Areas (LGAs) of Plateau State, Nigeria, between September 2019 and March 2020. It employed a cross-sectional study using snowball sampling. Herds containing three different ruminant groups that consented to participate in the study were chosen. The choice of snowball sampling was necessitated by the limited number of herders in these areas and due to the security challenges involved in accessing remote areas. The identified herders recommended more farms in the LGA that also fit the criteria. Because each herd includes a variety of strata, such as meat ruminants, lactating but dry ruminants, and lactating ruminants, the stratified random sampling approach was used to select which ruminants would be included in the study. Out of 349 ruminants, 95 were meat ruminants, 132 were dry female ruminants, while 122 lactating ruminants were sampled.

### Sample size determination

The sample size was calculated using the formula for estimating the prevalence of mastitis with a 95% confidence level and a 5% margin of error. Using the formula, N = Z^2^xP(1-P)/d^2,^where N is the minimum sample size, Z is the Z-statistic of 95% confidence level (1.96 for two-tailed tests), P is the prevalence of mastitis, and d is the allowable error margin of 5%. In this study, P was taken as 10.3% based on previous studies (Mbuk et al., 2016). By substitution, N was calculated to be 141.49, however, a total of 287 milk samples from ruminants were collected to ensure robustness of the study, account for both effects due to clustering, and the potential for nonresponse. About 213 milk samples were collected from 4 quarters of each of 54 lactating cows, except for one cow that had three blind teats, 68 milk samples collected from 34 does, and 34 ewes, respectively, and 12 fecal samples from pastoralists.

### California Mastitis Test

Individual ruminants were properly restrained and given a vet’s clinical inspection. Briefly, visual inspection and palpation were used to examine for clinical mastitis, while the CMT was used to further investigate the ruminants that did not have clinical mastitis but subclinical mastitis Ruminants that did not have clinical mastitis were subjected to further investigation for subclinical mastitis by using the CMT on milk samples from each half of the sampled ruminant. It was carried out by adding equal amounts of CMT reagent and milk from each half on the test paddle and was rocked for 10 seconds. Samples with a CMT score of 0 or T (trace) were considered negative, while those with CMT scores of 1 (mild clumping), 2 (moderate clumping), or 3 (heavy clumping) were considered positive for subclinical mastitis, according to (Anueyiagu et al., 2020).

### Sample collection

Following teat cleaning with 70% ethanol, aseptic methods were used to collect 10 mL of milk from each afflicted quarter or half of the ruminants’ udder, as well as samples of pastoralists’ feces (Zeryehun and Abera, 2017). Samples were transported in sterile screw-capped containers placed in cool boxes containing ice packs, maintaining a temperature of approximately 4 °C throughout the transport period.

Samples were transported to the Microbiology laboratory of the Federal College of Animal Health and Production Technology, Vom, within 4–6 hours of collection to ensure sample integrity. Human fecal samples of the pastoralists were taken in order to determine if there was any correlation between coliforms that would be isolated from ruminants and the pastoralists.

### Bacterial isolation and identification

Milk and fecal samples were first enriched in peptone water according to Geser et al. (2012) and incubated at 37 °C for 24 hours. Using the quadrant streaking technique, a loopful of broth culture was streaked on sterile MacConkey (Oxoid, UK) and Eosin Methylene Blue (EMB) agars (Oxoid, UK) and incubated at 37 °C for 24–48 hours. Lactose-fermenting colonies with characteristic morphology were subjected to Gram staining and a battery of biochemical tests (indole, methyl red, Voges-Proskauer, citrate utilization, oxidase, and catalase) for presumptive identification of coliforms.

The confirmatory screening was done using Oxiod Microbact GNB 24E following the manufacturer’s recommendations on presumptive Gram-stained coliforms. From an 18–24-hour culture, one to three isolated colonies were selected, emulsified in 5.0ml of sterile saline, and vigorously mixed to obtain a homogeneous suspension. The plate containing the substrates was put in the holding tray, and 4 drops (about 100 µl) of the bacterial suspension were added using a sterile Pasteur pipette. Except for well 20, which was used to detect oxidase-positive and other Gram-negative bacilli, the substrates underlined in the wells were covered with sterile mineral oil. Results were read following the manufacturer’s instructions after an 18–24 hour incubation period at 37 °C. The steps of the method were carried out in the order that Balows et al. (1991) recommended.

A prepared suspension of isolates to a turbidity equivalent to 0.5 McFarland standards was placed on Brilliance ESBL Chromogenic Culture Medium (Oxoid, UK) following the report by Ezeanya et al. (2017). At 37 °C between 24 hours and 48 hours, inoculated plates were incubated aerobically; color changes of colonies were observed and interpreted according to Oxoid, UK guidelines. The positive control used was *Klebsiella pneumoniae* ATCC 700603, while the negative control was *E. coli* ATCC 25922. Confirmed ESBL-producing coliform isolates were stored in glycerol stock at –20 °C for further analysis.

### Molecular detection of ESBL genes

Genomic DNA was extracted from phenotypically confirmed ESBL-producing isolates using the boiling lysis method. PCR was conducted to amplify common ESBL-encoding genes, including *bla*_TEM_, and *bla*_CTX-M_, using gene-specific primers: F-TCCGCTCATGAGACAATAACC, R-TTGGTCTGACAGTTACCAATGC (Kiratisin et al., 2008); and F-CGCTTTGCGATGTGCAG, R-ACCGCGATATCGTTGGT (Villegas et al., 2008), respectively. PCR products were visualized by gel electrophoresis on 1.5% agarose gels stained with ethidium bromide. Molecular weights were compared against a 931 bp, 550 bp and 868 bp for *bla*_TEM_, *bla*_SHV_, and *bla*_CTX-M_ DNA ladders. Positive and negative control strains were included in each run.

### Phylogeny

Phylogenetic relationships were inferred using the maximum likelihood method based on the Jukes-Cantor model. The corresponding taxa’s percentage of clustered trees is displayed next to the branches. The initial tree(s) for the heuristic search were constructed using the Neighbor-Joining method based on a matrix of pairwise distance calculated with the Maximum Composite Likelihood approach. With branch lengths expressed as the number of substitutions per site, the tree is drawn to scale. The analysis involved 36 nucleotide sequences. Evolutionary analyses were conducted in MEGA v.6.06 (Tamura et al., 2013) with bootstrap replicate values set at 1,000.

### Ethical considerations

Ethical approval was obtained from the Animal Ethics Committee of NVRI, Vom with reference number NVRI/AEC/02/64/19. Ethical approval for the collection of fecal samples from pastoralists was obtained from the Health Research Ethics Committee of Plateau Specialist Hospital, Jos, Plateau State, under reference number NHREC/09/23/2010b.

### Statistical analysis

Data were entered and analyzed using Microsoft Excel and R Commander version 2.10-0. Descriptive statistics such as frequencies and percentages were computed for relevant variables. Inferential statistics were applied to determine associations between variables. Chi-square tests were used to compare proportions of pathogen detection across the groups, and a p-value < 0.05 was considered statistically significant. Results were presented using tables and graphs for clarity and ease of interpretation.

## Results

In Plateau State, 287 milk samples were taken from 54 cows, 34 ewes, and 34 does. Of them, the CMT revealed that 79 (27.5%) had subclinical mastitis. Among the ruminant species with SCM, the prevalence rates were 18 (22.8%) in does, 23 (29.1%) in ewes, and 38 (48.1%) in cows. The prevalence of SCM varied significantly among the ruminant groups, according to statistical analysis (χ² = 8.23, p = 0.0163), suggesting that species type affected the chance of SCM occurrence (**Table 1**).

**Table 1:** Prevalence of SCM in ruminants in Plateau State.

Ruminants	No of samples	CMT-positive (%)	95% CI	χ^2^	p-value
Cow	213	38 (17.8)	13.0-23.7	8.23	0.0163
Ewes	68	23 (33.8)	22.3-46.4		
Does	68	18 (26.5)	16.2-38.9		
Total	287	79 (27.5)	22.5-33.0		

*E. coli* was the most commonly isolated coliform among the 79 CMT-positive samples, appearing in 50 (63.3%) of them. *Citrobacter freundii* (6.3%), *K. pneumoniae* (21.6%), *K. oxytoca* (2.5%), and *K. aerogenes* (6.3%) came next. *E. coli* was most prevalent in cows (71%), followed by ewes (60.9%) and does (50%), according to the distribution of these germs among the ruminant species. Notwithstanding these variations, the Fisher’s exact tests for the bacteria showed only *C. freundii* had a marginal statistically significant difference in the prevalence of coliform across the ruminant groups (**Table 2**). *E. coli* was isolated from 4 out of the 12 fecal samples of pastoralists examined.

**Table 2:** Occurrence of coliforms isolated from CMT-positive milk samples.

Ruminants	No of CMT-positive samples (%)	Isolate (%)				
		*E. coli*	*K. pneumoniae*	*K. oxytoca*	*C. freundii*	*K. aerogenes*
Cows	38(48.1)	27(71.0)	7(18.4)	2(5.3)	0(0.0)	2(5.3)
Ewes	23(29.1)	14(60.9)	6(26.1)	0(0.0)	3(13.0)	0(0.0)
Does	18(22.8)	9(50.0)	4(22.2)	0(0.0)	2(11.1)	3(16.7)
Total	79 (100)	50(63.3)	17(21.6)	2(2.5)	5(6.3)	5(6.3)
Fisher’s p-value		0.398	0.872	0.217	0.050*	0.098

It was determined that 42 (53.2%) of the 79 CMT-positive samples were coliform isolates that produced ESBLs. The most common isolate that produced ESBLs was *E. coli* (50%), which was followed by *K. pneumoniae* (21.4%), *K. aerogenes* (16.7%), *C. freundii* (9.5%), and *K. oxytoca* (2.4%). These isolates were found in all three ruminant species, with the ESBL-positive pool consisting of cows (16), ewes (18), and does (8) (**Table 3**).

**Table 3:** Occurrence of ESBL-producing coliforms in ruminants and pastoralist and their respective genes in ruminants with SCM.

Ruminants	No. of positive CMT samples	ESBL genes sampled	Livestock															χ 2		p-value
			*E. coli*			*K. pneumoniae*			*K. oxytoca*			*C. freundii*			*K. aerogenes*					
			%	*bla*_TEM_	*bla*_CTX_	%	*bla*_TEM_	*bla*_CTX_	%	*bla*_TEM_	*bla*_CTX_	%	*bla*_TEM_	*bla*_CTX_	%	*bla*_TEM_	*bla*_CTX_			
Cows	38 (48.1)	16	9 (12.5)	1	1	3 (18.8)	0	4	1 (6.3)	1	2	0 (0.0)	1	2	3 (18.8)	1	0			
Ewes	23 (29.1)	18	8 (44.4)	1	1	4 (22.2)	0	2	0 (0.0)	0	0	3 (16.7)	0	0	3 (16.7)	0	1			
Does	18 (22.8)	8	4 (50.0)	2	2	2 (25.0)	1	2	0 (0.0)	0	0	1 (12.5)	0	0	1 (12.5)	0	0			
Total	79 (100)	42	21 (50.0)	4	4	9 (21.4)	1	8	1 (2.4)	1	2	4(9.5)	1	2	7 (16.7)	1	1			
Pastoralists																				
Pastoralists	NA	12	4 (33.3)	4	0	0	1	0	0	0	0	0	0	0	0	0	0			

**Figure 1:**
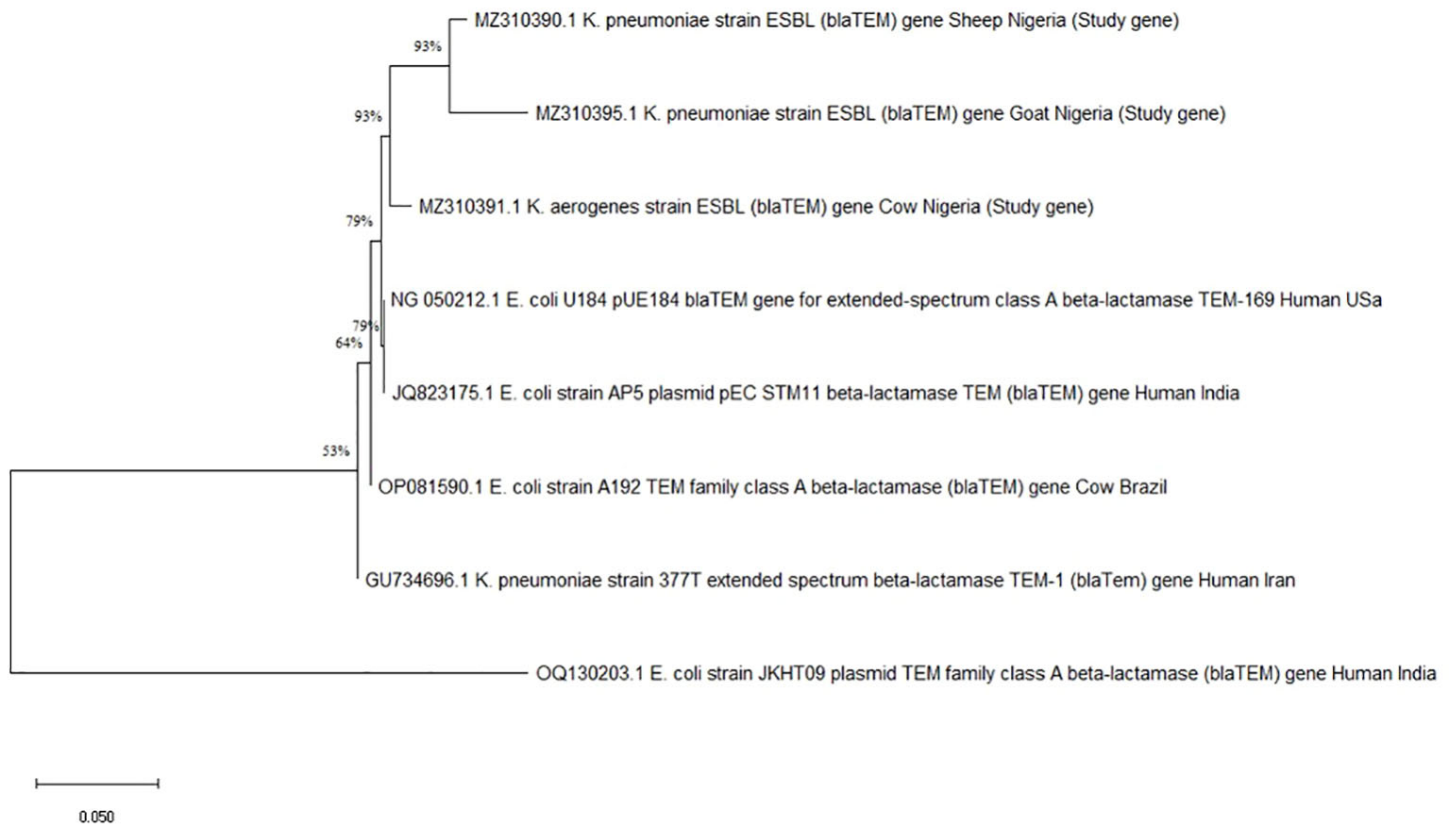
Phylogenetic tree showing genetic relationships based on the *bla*_TEM_ gene. Each branch represents a gene, identified by names and origins, with percentage values indicating bootstrap support for the nodes.

**Figure 2:**
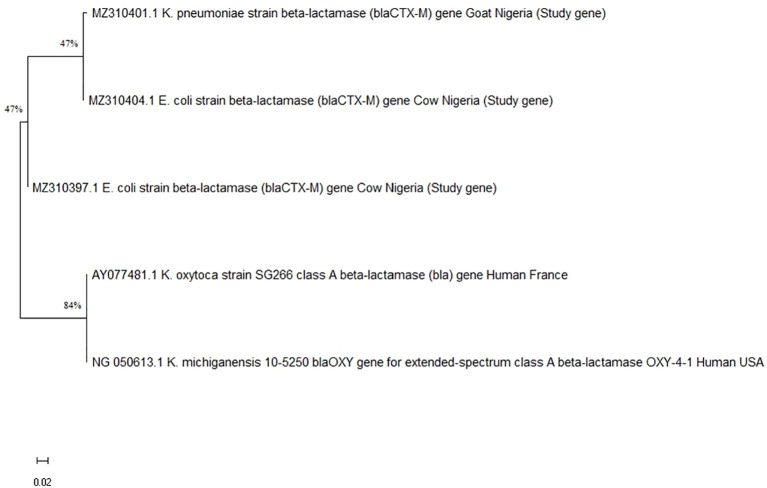
Phylogenetic tree showing the genetic relationship of various beta-lactamase genes. Branches indicate strains: *K. pneumoniae* from goat, *E. coli* from cows, and *K. oxytoca* and *K. michiganensis* from humans, with bootstrap values of fortyseven and eighty-four percent.

Two important ESBL genes, *bla*_TEM_ and *bla*_CTX-M_, were found in 46 coliform isolates by PCR analysis. Among all coliform species and ruminant sources, the *bla*_TEM_ gene was more common. *K. pneumoniae* and *K. aerogenes* from cows and does reveal dual carriage of both genes in several isolates, while *E. coli* isolates from pastoralists (4 samples) demonstrated 100% carriage of the *bla*_TEM_ gene with no detection of *bla*_CTX-M_ (**Table 3**). Using the Maximum Likelihood Method, phylogenetic analysis further verified the *bla*_TEM_ (**Figure 1**) and *bla*_CTX-M_ genes’ (**Figure 2**) genetic relatedness across species.

## Discussion

In this study, we identified ESBL-producing microorganisms implicated in SCM among ruminants, some of which are known to possess zoonotic potential. While direct transmission from animals to humans was not assessed, our findings contribute to the growing body of evidence suggesting that individuals in close contact with livestock may be at increased risk of exposure to antimicrobial-resistant pathogens and resistance genes (Agusi et al.., 2024). This underscores the importance of continued surveillance within a One Health framework (Aworh et al., 2019; Johnson et al., 2007).

This study showed that subclinical mastitis in ruminants was more prevalent than clinical mastitis. This is attributed to the subtle nature of SCM, making it less problematic for farmers as it scarcely affects the physical quality of milk; hence, farmers focus more on clinical mastitis since subclinical mastitis is difficult to detect. As a result, untreated subclinical mastitis festers and has the potential to spread from animal to animal. Cows, Ewes, and Does have a prevalence of 38%, 23%, and 18%, respectively. These figures corroborate earlier findings by Gebrewahid et al. (2012), who reported a prevalence of 18.03% for Does but a higher prevalence of 28.14% for Ewes. This may be connected to Nigeria’s prevalent poor environmental hygiene and farm management practices, which tend to increase the risk of infectious agents spreading among herds in feedlots and during transhumance, a common practice among local pastoralists. In this study, 79 ruminant milk samples with SCM contained 50 coliform isolates identified as *E. coli, K. pneumoniae, K. oxytoca, C. freundii*, and *K. aerogenes*. This is in close agreement with Garedew et al. (2012), who identified the primary mastitogens as *E. coli, Klebsiella* spp.*, Enterobacter* spp.*, Citrobacter, Serratia*, and *Proteus*. Organic materials, such as bedding and manure, typically contain large amounts of coliform bacteria (environment). Therefore, from an epidemiologic perspective, environmental factors accounted for the majority of pathogen infections in this study. When coliform bacteria come into contact with teat ends, they enter the udder through the teat sphincter. Coliform bacteria either proliferate quickly or go dormant after entering the mammary gland.

*E. coli* had the highest prevalence rate of 63.3% among all the ruminants sampled, followed by *K. pneumoniae* with a prevalence of 21.6%, and *K. aerogenes* with a prevalence of 6.3%. This might be because *E. coli* is a common commensal organism in the gastrointestinal tract of ruminants and is more readily shed in feces, making it more likely to be isolated during sampling, especially in environments with suboptimal hygiene and management practices. In a cross-sectional study comparable to this one, 54 distinct bacterial species were found in Gondar, Ethiopia. However, the frequently isolated Gram-negative bacterial pathogens were *E. coli* (29.6%), *Pseudomonas aeruginosa* (18.5%), and *K. pneumoniae* (16.7%) (Garedew et al., 2012).

ESBL-producers strains identified in this study carried either *bla*_CTX-M_ or *bla*_TEM_. The overall prevalence rate of *bla*_CTX-M_ and *bla*_TEM_ in ruminant mastitis was 36.96% and 26.08%, respectively. Ali et al. (2016) reported that *bla*_CTX-M_ was the predominant ESBL gene detected from bovine mastitis, with a higher prevalence of 77.78% isolates. However, Yu et al. (2019) had a contrary result where *bla*_TEM_ was the most frequently detected resistance gene with 83.1% prevalence in bovine mastitis, followed by *bla*_CTX-M_ with 66.3% prevalence.

The resistance gene, *bla*_TEM,_ has increasingly been identified in many different sources, including humans, animals, and the environment. During the last decade, it has virtually displaced the other ESBLs within Enterobacteriaceae (Canton et al., 2012). Among 12 stool samples collected from pastoralists, only four *E. coli* were isolated, and all were *bla*_TEM_. This is significant since it reflects AMR in human isolates, and this may reflect likely transmission of resistant strains or resistance genes from humans to animals or vice versa, particularly in environments with close animal-human contact. Despite the small sample size, this raises concerns about the likely reservoir of resistance genes among the population in contact with livestock.

According to phylogenetic analysis, some of the *bla*_CTX-M_ and *bla*_TEM_ gene sequences from the ruminants in this study shared ancestry with human genes from various geographical locations. Due to the high level of phylodiversity found in ruminants, clones with different genetic backgrounds may be responsible for the transmission of coliforms carrying the *bla*_CTX-M_ and *bla*_TEM_ genes (Jena et al., 2017). Surprisingly, none of the samples screened in this study revealed the presence of the *bla*_SHV_ gene. This trend was found in Yang et al., 2018 where *bla*_SHV_ was not detected, while *bla*_CTX-M_ and *bla*_TEM_ had 97.26% and 71.23% of isolates in cows with mastitis. The absence of *bla*_SHV_ genes in this study may be due to the difference in type and volume of consumption of antibiotics and the difference in time in which the isolates were collected (Al-Agamy et al., 2009). Also, it has become evident that once a *bla*_CTX-M_ type enters an area, it becomes prevalent, replacing *bla*_TEM_ and *bla*_SHV_ as the dominating ESBL (Canton et al., 2012).

This study highlights the One Health implications of ESBL-producing coliforms in Nigerian ruminants with SCM. The veterinary, medical, and environmental sectors must work together to address this issue in order to stop the spread of resistance and protect animal and human populations.

## Conclusion

This study emphasizes the zoonotic threat posed by (ESBL)-producing coliform bacteria in Nigeria as well as the public health importance of SCM in ruminants. The hidden but significant danger of AMR transmission from animals to people is highlighted by the high prevalence of ESBL-producing *E. coli* and *Klebsiella* sp*ecies*, especially those carrying the *bla*_TEM_ and *bla*_CTX-M_ genes, in otherwise healthy nursing animals.

There is a significant risk of zoonotic spillover of these resistant infections due to the informal selling and consumption of raw milk, as well as the frequent close human-animal contacts found in Nigeria’s pastoral and smallholder dairy systems. According to the findings, a One Health strategy that encourages better farm hygiene, prudent antibiotic usage, integrated surveillance, and livestock handler education should be put into place.

In the end, combating ESBL-producing organisms in animal health systems is crucial for maintaining the efficacy of vital antibiotics for future generations as well as for protecting animal productivity and reducing the growing threat of AMR to human health.

## Data Availability

The original contributions presented in the study are included in the article/supplementary material, further inquiries can be directed to the corresponding authors.
